# Piperlongumine Induces Apoptosis and Cytoprotective Autophagy via the MAPK Signaling Pathway in Human Oral Cancer Cells

**DOI:** 10.3390/biomedicines11092442

**Published:** 2023-09-01

**Authors:** Eun-Young Choi, Eun-Ji Han, Su-Ji Jeon, Sang-Woo Lee, Jun-Mo Moon, Soo-Hyun Jung, Ji-Youn Jung

**Affiliations:** 1 Department of Companion and Laboratory Animal Science, Kongju National University, Yesan-gun 32439, Republic of Korea; secure801@gmail.com (E.-Y.C.); suranhan910@gmail.com (E.-J.H.); suzy4099@gmail.com (S.-J.J.); leesw9748@gmail.com (S.-W.L.); moonjunmo541@gmail.com (J.-M.M.); jsh0019y@gmail.com (S.-H.J.); 2 Research Institute for Natural Products, Kongju National University, Yesan-gun 32439, Republic of Korea

**Keywords:** piperlongumine, oral cancer, apoptosis, autophagy, MAPK pathway, anti-cancer agent

## Abstract

Oral cancer is a malignant tumor that primarily affects areas such as the lips, tongue, buccal mucosa, salivary gland, and gingiva and has a very high malignancy. Piperlongumine (PL), isolated from long pepper (*Piper longum* L.), is a natural alkaloid with pharmacological effects, such as anti-inflammatory and anti-atherosclerotic effects. The effect and mechanism of PL in oral cancer cell lines has not been explored. Therefore, this study aimed to investigate the mechanism of anticancer effects of PL in the human oral cancer cell lines MC-3 and HSC-4 in vitro. This study demonstrated that PL inhibits cell proliferation by inducing apoptosis and autophagy in human oral cancer cell lines, which was confirmed by the levels of apoptosis- and autophagy-related proteins through Western blotting. Moreover, the pharmacological blockade of autophagy activation by hydroxychloroquine (HCQ), an autophagy inhibitor, significantly improved PL-induced apoptosis in MC-3 cells, suggesting a cytoprotective effect. In addition, activation of the mitogen-activated protein kinase (MAPK) signaling pathway contributed to PL-induced apoptosis. Collectively, the study suggested that combining an autophagy inhibitor with PL treatment can exert effective anticancer properties in oral cancer cells by inducing apoptosis and cytoprotective autophagy via the JNK-mediated MAPK pathway.

## 1. Introduction

Cancer is a widespread disease characterized by uncontrolled cell differentiation, which results in malignant tumors attacking both nearby and distant body sites via lymphatic vessels/bloodstream [1]. Oral cancer is a severe health problem worldwide, with 275,000 new cases each year, making it the sixth most prevalent type of cancer globally [2,3]. Oral cancer is a malignant tumor that primarily affects areas such as the lips, tongue, buccal mucosa, salivary gland, and gingiva; it has a low incidence compared to carcinomas in other areas, but a very high malignancy [4]. Oral cancer treatment methods include surgery, radiotherapy, and chemotherapy; however, there are numerous challenges regarding post-treatment progress, or side effects, such as damage to normal cells and decreased immunity. The 5-year survival rate of patients with oral cancer remains low despite new advances in treatment and diagnosis [5,6]. Thus, developing a new treatment method using natural substances with few side effects is critical for inhibiting tumor proliferation and migration and improving the survival rate and quality of life of such patients.

Piperlongumine (PL), a natural alkaloid extracted from long pepper (*Piper longum* L.), has numerous biological activities, including anti-inflammatory, anti-atherosclerotic, anti-platelet, anti-depressive, anti-bacterial as well as analgesic effects [7,8,9,10,11]. PL is conventionally used to treat diseases, such as malaria, viral hepatitis, and gastrointestinal and general respiratory problems [12]. It exhibits anticancer effects against breast cancer [13], colorectal cancer [14,15], gastric cancer [16], lung cancer [17,18], and prostate cancer [19] by selectively inducing apoptosis in cancer cells without damaging normal cells and tissues. In contrast, although PL is known to have anticancer effects on several types of cancer cells, its effect on apoptosis induction and its mechanism of action along with autophagy have not yet been determined in oral cancer cell lines. Therefore, elucidation of the molecular mechanisms underlying the anticancer effects of PL in oral cancer was considered necessary.

Apoptosis is a highly organized process regulated by cellular proteins via a series of signaling cascades [20]. It removes damaged, virus-infected, and cancer cells [21]. Apoptosis contributes to various diseases, including cancer and septic shock [22]. Since apoptosis causes structural changes in cells, distinct morphological features, such as DNA fragmentation, pyknosis, and karyorrhexis, are observed [23]. Pro-apoptotic proteins, namely Bcl-2-associated X (Bax) and Bcl-2 homologous antagonist killer (Bak), form oligomers and release cytochrome c from the mitochondria, thereby activating the caspase cascade and inducing apoptosis [24]. In contrast, anti-apoptotic proteins, namely B-cell lymphoma 2 (Bcl-2) and B-cell lymphoma-extra-large (Bcl-xL), bind to and inactivate pro-apoptotic proteins [25]. Excessive expression of anti-apoptotic proteins causes an imbalance between apoptosis-related proteins, which in turn inhibits apoptosis. Hence, the regulation of pro- and anti-apoptotic proteins plays a crucial role in overcoming drug resistance in cancer cells [26].

Autophagy is the most critical degradation process for cell homeostasis and survival. A series of essential autophagy-related proteins primarily regulate the basic steps involved in autophagosome formation. A microtubule-associated protein, namely 1A/1B-light chain 3 (LC3), is one of the most important regulators for the formation of autophagosomes [27]. Consequently, phosphatidylethanolamine binds to LC3-I through ubiquitination, forming autophagosome-bound form, LC3-II, and analyzing this conversion to LC3-II reflects the process of autophagy [28]. Mammalian target of rapamycin (mTOR) integrates various stimulatory and signaling networks to stimulate and translate the synthesis of proteins, lipids, and nucleotides, blocking catabolic processes, such as autophagy, at the transcriptional level [29]. Therefore, the transformation of LC3-I to LC3-II and the downregulation of mTOR are vital factors for evaluating autophagic activity. Numerous compounds in plant extracts are known to induce both autophagy and apoptosis, and autophagy plays a cytoprotective role by inhibiting apoptosis [30]. Whether autophagy exhibits a cytotoxic or cytoprotective effect in cancer cells remains controversial, since autophagy is known to induce apoptosis under certain stress conditions [31]. Thus, determining whether autophagy activation by chemotherapeutic agents has a cytoprotective or cytotoxic effect on cancer cells is critical.

Mitogen-activated protein kinase (MAPK) is a serine/threonine kinase involved in cellular responses to stress, differentiation, proliferation, and also cell death [32]. The main MAPK pathways include extracellular signal-regulated kinase (ERK), c-Jun N-terminal kinase (JNK), and p38, each responding to various cell stimuli. The activation of ERK is involved in the growth, proliferation, and survival of cancer cells, whereas the JNK and p38 pathways respond primarily to stresses such as oxidative stress and irradiation and are involved in cytokine production, metabolism, inflammation, and apoptosis [33,34]. In cancer therapy, targeting these signaling molecules can be critical for drug development and cancer research.

The present study aimed to investigate the effects of PL on the viability of MC-3 and HSC-4 cells, both human-derived oral cancer cell lines, and understand their underlying biological mechanisms. The study also aimed to determine whether apoptosis and autophagy could be simultaneously induced via the MAPK pathway by investigating the effect of PL on apoptosis and autophagy. In addition, the cytoprotective effects of PL-induced autophagy in MC-3 cells were investigated.

## 2. Materials and Methods

### 2.1. Materials and Reagents

**Figure 1:**
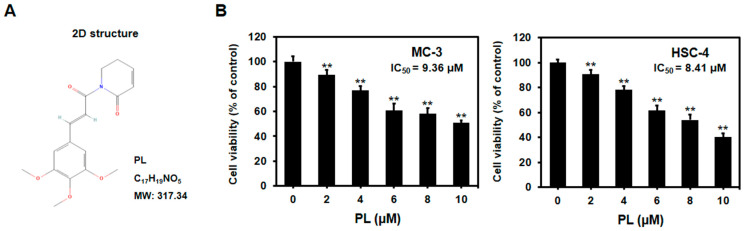
Anti-proliferative and cytotoxic effect of PL in oral cancer cell lines. (**A**) The chemical structure of PL (Source: PubChem CID: 637858). (**B**) MC-3 and HSC-4 cells were treated with indicated concentrations of PL for 24 h, and cell viability was detected by MTT assay. The results are representative of three independent experiments (Statistically significant differences, ** *p* < 0.01 vs. the control group).

PL (Figure 1A; purity ≥ 97%) was purchased from Aladdin Chemical Co., Ltd. (Shanghai, China). 4′,6-Diamidino-2-phenylindole (DAPI), dimethyl sulfoxide (DMSO), 3-(4,5-dimethylthiazol-2-yl)-2,5-diphenyltetrazolium bromide (MTT), JNK inhibitor SP600125, and general reagents were purchased from Sigma-Aldrich (St. Louis, MO, USA). A stock solution of PL was prepared in DMSO at 10 μM, stored at −20 °C. The control group was treated with DMSO alone. The Fluorescein Isothiocyanate (FITC) Annexin V apoptosis detection kit was obtained from BD Pharmingen™ (San Diego, CA, USA). Bax, poly (ADP-ribose) polymerase (PARP), ERK1/2, p-ERK1/2, JNK, p-JNK, p38, p-p38, LC3, Beclin-1, p-mTOR, and secondary antibody rabbit IgG were obtained from Cell Signaling Technology (Beverly, MA, USA). Bcl-2 was purchased from Bioworld Technology (Qixia, Nanjing, China). Secondary antibody mouse IgG and β-actin were purchased from Santa Cruz Biotechnology Inc. (Dallas, TX, USA). 3-Methyladenine (3-MA) and hydroxychloroquine (HCQ) were obtained from Selleck Chemical (Houston, TX, USA).

### 2.2. Cell Culture

Oral cancer cell lines MC-3 and HSC-4 were provided by Professor Sung-Dae Cho (Seoul National University, Seoul, Korea), thawed, and cultured in a 75 cm^2^ cell culture flask. Cells were cultured in DMEM medium (Welgene, Gyeongsan, Republic of Korea) containing 5% fetal bovine serum (FBS; Grand Island, NY, USA) and 1% streptomycin/penicillin (Gibco BRL, Grand Island, NY, USA). Cells were cultured in a 5% CO_2_ incubator maintained at 37 °C. The medium was changed every 2–3 days. When the flask was approximately 80–90% full of cells, it was washed with phosphate-buffered saline (PBS; Biosesang, Seongnam, Republic of Korea) and subcultured with trypsin-EDTA (Gibco, Grand Island, NY, USA).

### 2.3. Cell Viability Assay

MTT assay was performed to observe cytotoxicity by PL in oral cancer cells. The cells were added to a 96-well plate at a density of 2 × 10^4^ cells/mL and incubated at 37 °C in the presence of 5% CO_2_ for 24 h. Thereafter, the cells were treated with various concentrations of PL (0, 2, 4, 6, 8, and 10 μM) and were further incubated for 24 h. SP600125 (50 μM) and HCQ (20 μM) were pre-treated for 2 h before PL treatment. Next, after the removal of the medium, the cells were treated with 40 μL of the MTT solution and incubated for 2 h. After removing the MTT solution, this was followed by the addition of 100 μL of DMSO to facilitate the dissolution of the formazan crystals formed therein. Then, the absorbance at 595 nm was measured using an ELISA reader (Bio-Rad Laboratories, Hercules, CA, USA).

### 2.4. DAPI Staining

Characteristic morphological changes of nuclei during apoptosis were observed by DAPI staining. Cells incubated in a 60 mm dish at a density of 2 × 10^5^ cells/mL for 24 h were treated with PL (0, 4, and 8 μM) and maintained in a 5% CO_2_ incubator for 24 h. After removing the medium containing PL, the cells were washed with PBS and fixed with 4% paraformaldehyde for 15 min. After the fixation process, the cells were washed with PBS and treated with 2 mL of DAPI solution for observation under a fluorescence microscope (Zeiss AG, Thornwood, NY, USA). Apoptotic cells were counted as the ratio of DAPI-positive/total cells in four random fields.

### 2.5. Annexin V/PI Staining

The degree of apoptosis induced by PL in oral cancer cells was quantitatively analyzed via annexin V/PI staining, followed by assessment via fluorescence-activated cell sorting (FACS). The MC-3 and HSC-4 cells were treated with PL (0, 4, and 8 μM) and incubated in a CO_2_ incubator for 24 h. The cells were suspended in trypsin-EDTA and centrifuged at 300× *g* for 5 min to obtain cell pellets. The pellets were washed with ice cold PBS, centrifuged, and resuspended in 1xbinding buffer at a density of 2 × 10^5^ cells/mL. Annexin V and propidium iodide (PI) were treated and incubated for 15 min, and then measured by using a BD FACSCalibur™ flow cytometer (BD Biosciences, Franklin Lakes, NJ, USA).

### 2.6. Acridine Orange Staining

Acridine orange staining was performed to confirm the presence of acidic vesicular organelles (AVOs), a characteristic of autophagy. After 24 h of incubation in a 60 mm dish (cell density: 2 × 10^5^ cells/mL), the cells were treated with PL (0, 4, and 8 μM) and incubated in a CO_2_ incubator for another 24 h. Subsequently, the medium containing PL was removed and the cells were washed twice with PBS. Next, the cells were fixed with 4% paraformaldehyde for 15 min. They were washed twice with PBS to remove paraformaldehyde and treated with 2 mL of acridine orange solution (5 μg/mL, 10 min) for observation under a fluorescence microscope.

### 2.7. Western Blotting

Western blotting was performed to investigate the expression of apoptosis and autophagy-related proteins according to PL treatment. MC-3 and HSC-4 cells were cultured in a 75 cm^2^ flask and maintained in a CO_2_ incubator for 24 h. After incubation, the medium was removed, and fresh medium containing PL (0, 4, and 8 μM) was treated and incubated for an additional 24 h. The cells were subsequently suspended in trypsin-EDTA and were centrifuged at 300× *g* at 4 °C for 5 min. The resultant cell pellet was subjected to treatment with cell lysis buffer, incubated at 4 °C for 20 min, and centrifuged at 15,920× *g* and 4 °C for 5 min, and the supernatant was used as the cell lysate. The extracted protein was quantified using the Bradford protein assay. The proteins were isolated in 12% sodium dodecyl sulfate-polyacrylamide gel electrophoresis (SDS-PAGE) and then transferred to a nitrocellulose membrane (Bio-Rad, CA, USA). Bovine serum albumin (BSA) and 5% skim milk was used for blocking the membranes 2 h. Primary antibody (1:1000) was incubated at 4 °C overnight. Thereafter, secondary antibody (1:1000 or 1:2000) incubation was conducted for 2 h. An ECL detection reagents (Pierce, Rockford, IL, USA) was used to visualize the protein bands, which were quantified by using ImageJ Software Version 1.52a (provided by NCBI).

### 2.8. Statistical Analysis

All experiments were repeated at least three times, and the results are presented as mean ± standard deviation (SD). Comparisons among multiple groups were determined by one-way ANOVA followed by Dunnett’s test. The difference between two groups were analyzed by Student’s *t*-test. The data were evaluated with IBM SPSS Statistics Version 27 (Armonk, NY, USA); *p* < 0.05 was regarded as statistically significant.

## 3. Results

### 3.1. PL Reduced Cell Viability of Human Oral Cancer Cells

MTT assay was performed to determine the effect of PL on the viability of oral cancer cells. The cells were first incubated with different concentrations of PL (0, 2, 4, 6, 8, and 10 μM) for 24 h, followed by the MTT assay. PL was found to significantly reduce the survival of MC-3 and HSC-4 cells at 2 μM and higher concentrations (Figure 1B). In addition, the IC_50_ values of PL in MC-3 and HSC-4 cells were 9.36 μM and 8.41 μM, respectively. The results suggested that PL reduced the viability of oral cancer cells in a dose-dependent manner. Therefore, the MTT assay demonstrated that even at low doses, PL restricted the growth of MC-3 and HSC-4 cells. To simplify the study, 4 and 8 μM PL concentrations were selected for the subsequent experiments.

### 3.2. PL Induced Apoptosis in Human Oral Cancer Cells

**Figure 2:**
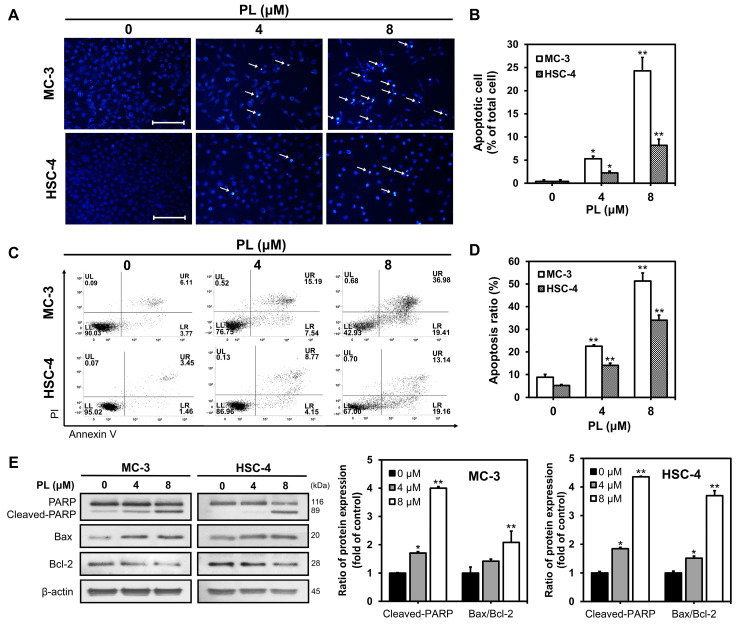
Induction of apoptosis in oral cancer cell lines upon PL treatment. (**A**) MC-3 and HSC-4 cells were seeded for 24 h and then treated with 0, 4, and 8 μM of PL for 24 h. After that, cells were stained with 1× DAPI solution and examined under fluorescence microscope (scale bar, 100 μm). The white arrows indicate chromatin condensations. (**B**) The bar graph represents the average of five fields under a fluorescence microscope, and the percentage of apoptotic cells among total cells. (**C**) MC-3 and HSC-4 cells were treated with 0, 4, and 8 μM of PL for 24 h, stained with annexin V/PI and analyzed by FACS. (**D**) The bar graph represents the apoptosis ratio (sum of early and late apoptosis). (**E**) Cells were harvested to measure the levels of PARP, Bax and Bcl-2 proteins by Western blotting in MC-3 and HSC-4 cells. β-actin was used as loading control. The results are representative of three independent experiments (statistically significant differences, * *p* < 0.05, ** *p* < 0.01 vs. the control group).

DAPI staining and flow cytometry were performed to determine whether the decrease in cell viability caused by PL, in the previous experiment, was due to apoptosis. DAPI staining was performed to quantify apoptotic cells. After incubating the cells with 0, 4, and 8 μM PL for 24 h, they were stained with DAPI to observe apoptotic bodies such as DNA fragments and condensed cytoplasm (Figure 2A). The number of apoptotic bodies increased to 0.3%, 5.2%, and 24.2%, respectively, in MC-3 cells, and to 0.3%, 2.2%, and 8.1%, respectively, in HSC-4 cells, in a dose-dependent manner (Figure 2B).

Annexin V/PI staining was performed to determine whether cell apoptosis was involved in the cytotoxic effects of PL (Figure 2C). The percentage of early/late apoptosis significantly increased when the cells were subjected to treatment with 4 and 8 μM PL compared to that in the control group, for both MC-3 and HSC-4 cells (Figure 2D). The results confirmed that the reduced viability of oral cancer cell lines, observed after PL administration, was due to apoptosis.

Western blot analysis was performed to examine the expression of apoptosis-related proteins in MC-3 and HSC-4 cells after PL treatment (Figure 2E). PARP, a protein related to DNA repair, was found to be fragmented, and expression of Bax, a pro-apoptotic protein, was found to be up-regulated. In contrast, the expression levels of Bcl-2, an anti-apoptotic protein, reduced with increasing PL concentrations in MC-3 and HSC-4 cells. The results suggested that PL induced apoptosis by increasing the expression of cleaved PARP and the ratio of Bax/Bcl-2.

### 3.3. PL Induced Apoptosis via MAPK Pathway in Human Oral Cancer Cells

**Figure 3:**
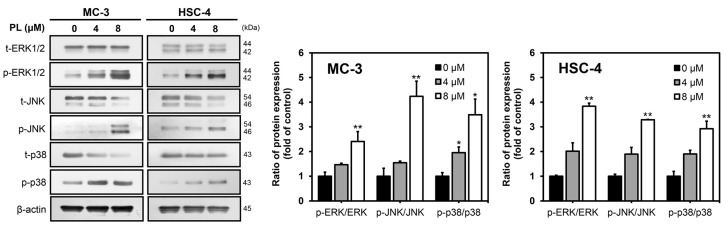
Effects of PL on the expression of the MAPK pathway-related proteins in oral cancer cell lines. MC-3 and HSC-4 cells were treated with indicated concentrations of PL for 24 h and harvested to measure expression levels of MAPK pathways; ERK, JNK, and p38 proteins were detected by Western blot analysis. β-actin was used as loading control. The results are representative of three independent experiments (statistically significant differences, * *p* < 0.05, ** *p* < 0.01 vs. the control group).

Western blotting was performed for ERK, JNK, and p38 proteins, which are major participants in the MAPK pathway, to determine the mechanism underlying apoptosis induction. Increased expression of p-ERK1/2, p-JNK, and p-p38 was observed in PL-treated cells than in the control group in both MC-3 and HSC-4 cell lines. The levels of t-ERK1/2, t-JNK, and t-p38 decreased in both the PL-treated cell lines (Figure 3). Protein quantification revealed that the level of phosphorylation increased with increasing PL concentrations in each cell type. The results collectively demonstrated that PL induced apoptosis by activating the MAPK signaling pathway.

### 3.4. PL Induced Autophagy in Human Oral Cancer Cells

**Figure 4:**
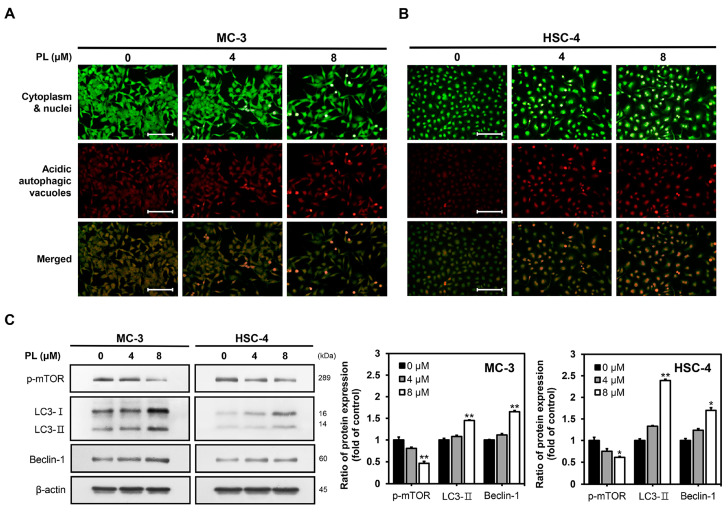
Effects of PL on the induction of autophagy in oral cancer cell lines. The (**A**) MC-3 and (**B**) HSC-4 cells were stained with acridine orange to confirm detection of AVOs and analyzed using a fluorescence microscope. The cytoplasm and the nucleus stained fluorescent green and the AVOs stained fluorescent red (scale bar, 100 μm). (**C**) Cells were harvested to measure the levels of autophagy-related proteins by Western blotting. β-actin was used as loading control. The results are representative of three independent experiments (statistically significant differences, * *p* < 0.05, ** *p* < 0.01 vs. the control group).

To investigate whether PL triggered autophagy in oral cancer cells, we first performed acridine orange staining to observe autolysosomes, which are typical for autophagy. The cells were stained with acridine orange and then compared with the control group. The PL-treated groups showed increased AVO formation in both MC-3 (Figure 4A) and HSC-4 (Figure 4B). Western blotting was performed to identify the expression of autophagy-related proteins, such as p-mTOR, Beclin-1, and LC3 (Figure 4C). The PL (4 and 8 μM)-treated groups showed a significant increase in Beclin-1 and LC3-II protein expression compared to the control group for each cell. In contrast, p-mTOR levels tended to decrease in a concentration-dependent manner. The results collectively demonstrated that PL induced autophagy in oral cancer cells.

### 3.5. PL Induced Cytoprotective Autophagy in Human Oral Cancer Cells

**Figure 5:**
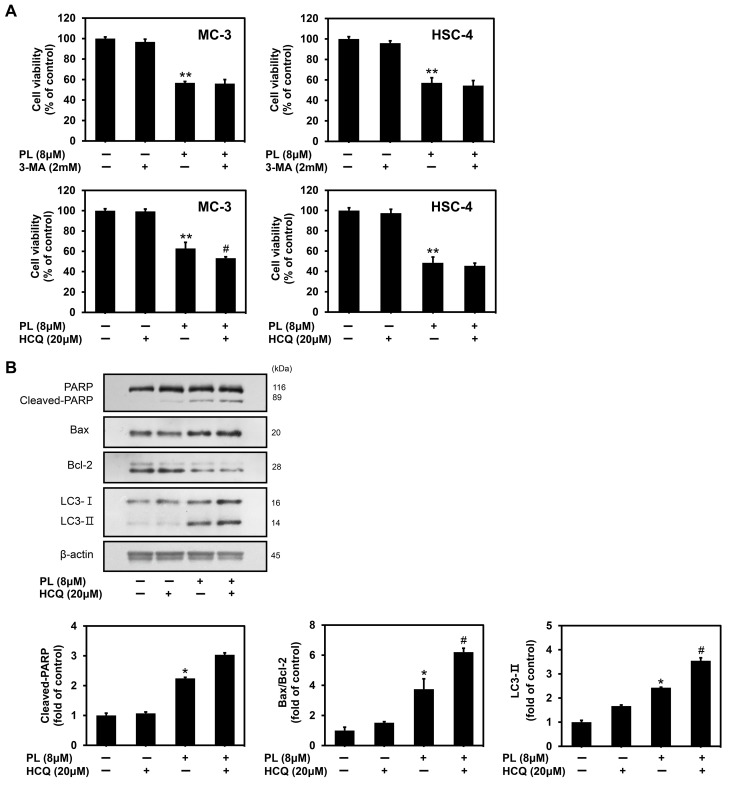
Induction of cell-protective autophagy in MC-3 oral cancer cells upon PL treatment. (**A**) The MC-3 and HSC-4 oral cancer cells were pretreated with 3-MA (2 mM) and HCQ (20 μM) for 2 h, followed by treatment with PL (0 and 8 μM) for 24 h. The cell viability was measure by MTT assay. (**B**) Protein levels of PARP, Bax, Bcl-2, LC3 were measured by Western blotting. β-actin was used as loading control. The results are representative of three independent experiments (statistically significant differences, * *p* < 0.05, ** *p* < 0.01 vs. the control group; ^#^ *p* < 0.05 vs. the PL treatment group).

Autophagy in cancer cells may have either cytotoxic or cytoprotective functions depending on the distinct cellular stresses and drug treatments. To determine the role of autophagy in the apoptosis process triggered by PL in oral cancer cells, we used the autophagy inhibitor 3-MA (early-stage inhibitor of autophagy) and HCQ (late-stage inhibitor of autophagy) to block the autophagy process. MTT assay confirmed that cell viability was greatly reduced in MC-3 cancer cells after PL treatment alone, which was consistent with our previous data (Figure 5A). On comparing the PL-treated group with that pretreated with 3-MA (2 mM) for 2 h, there was no significant difference in cell viability in both MC-3 and HSC-4 cell lines. However, when comparing the PL-treated group with that pretreated with HCQ (20 μM) for 2 h, the MC-3 cell viability was found to be decreased significantly, but there was no difference in HSC-4 cells. These results showed that the antitumor effects in oral cancer cells, especially MC-3 cells, induced by PL could be enhanced by HCQ. Therefore, to study the relationship between apoptosis and autophagy, the subsequent experiments were conducted with MC-3 cells using HCQ. Western blot analysis demonstrated that pretreatment with an autophagy inhibitor (HCQ) increased the expression of pro-apoptotic proteins (cleaved PARP and Bax) and decreased the expression of Bcl-2 compared to that with treatment with PL alone in MC-3 cells (Figure 5B). Furthermore, the accumulation of LC3-Ⅱ protein, induced by HCQ treatment, was confirmed. The results suggested that HCQ induced the formation of autophagosomes, accompanied by the inhibition of lysosomal degradation in MC-3 cells. Taken together, the results indicated that the combination treatment of PL with HCQ enhanced PL-induced apoptosis in MC-3 oral cancer cells. Therefore, autophagy was shown to play a protective role in MC-3 cells.

### 3.6. PL Regulated Apoptosis and Autophagy through the Activation of JNK

**Figure 6:**
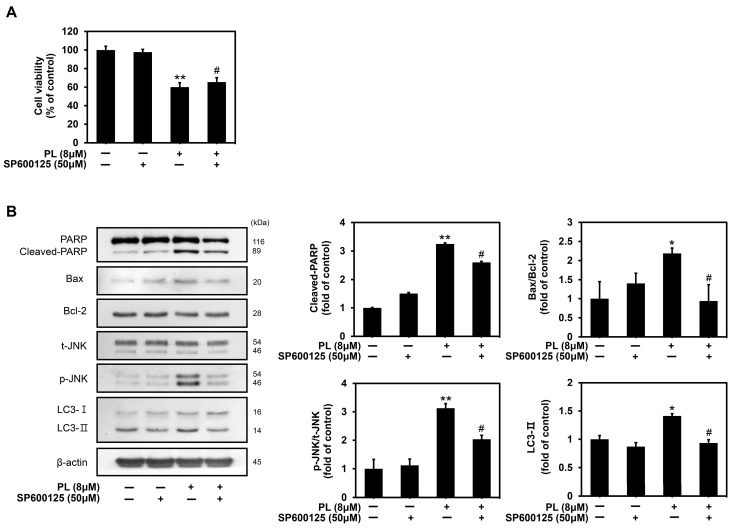
Regulation of apoptosis and autophagy through activation of JNK in MC-3 oral cancer cells upon PL treatment. (**A**) The MC-3 oral cancer cells were pretreated with SP600125, a JNK inhibitor (50 μM), for 2 h, followed by treatment with PL (0 and 8 μM) for 24 h. The cell viability was measure by MTT assay. (**B**) Protein levels of PARP, Bax, Bcl-2, t-JNK, p-JNK, LC3 were measured by Western blotting. β-actin was used as loading control. The results are representative of three independent experiments (Statistically significant differences, * *p* < 0.05, ** *p* < 0.01 vs. the control group; ^#^ *p* < 0.05 vs. the PL treatment group).

To elucidate the underlying mechanism of apoptosis induction, we used SP600125, a JNK inhibitor, to clarify the role of JNK in PL-induced apoptosis and autophagy. As shown in Figure 6A, JNK inhibition by SP600125 increased the viability of MC-3 cells. Western blot analysis demonstrated that JNK inhibition by SP600125 inhibited PL-induced apoptosis and autophagy in MC-3 cells (Figure 6B). The results collectively indicated that PL-induced apoptosis and autophagy are regulated by JNK activation.

## 4. Discussion

Despite new advances in treatment and diagnosis, the 5-year survival rate of patients with oral cancer remains low [6]. The chemotherapeutic agents currently available for various tumors have been reported to exert no significant anticancer effect owing mainly to adverse side effects, systemic organ toxicity, and drug resistance in tumors [35]. Thus, the identification of natural compounds with promising anticancer activities and tolerable side effects would be vital for improving the survival rate and quality of life of patients with oral cancer. PL is a well-known natural alkaloid found in the roots of *Piper longum* L. (long pepper) and has numerous biological activities, including anticancer effects [12]. In particular, combining anticancer drugs with natural compounds would be a potential strategy for improving the effects of chemotherapy on cancer cells [36]. Interestingly, although PL is toxic to various cancer cells, it rarely damages normal human cells, thereby indicating its selectivity for cancer cells over normal human cells [37]. A previous study reported that PL increases the cellular level of reactive oxygen species (ROS) and selectively induces apoptosis in cancer cells with no apparent toxicity in normal cells [38]. However, very few studies have examined the effects of PL on the induction of apoptosis and autophagy in human cancer cell lines and their underlying molecular mechanisms. Accumulating evidence has suggested that several chemotherapeutic agents and anticancer drugs can induce autophagy in various cancer cell lines, and that targeted autophagy may be a promising strategy for cancer treatment [39]. Thus, understanding the relationship between apoptosis and autophagy could provide new strategies for cancer treatment [40]. The current study investigated the effects of PL on cell viability, apoptosis, and autophagy, as well as the mechanisms of anticancer action in the oral cancer cell lines MC-3 and HSC-4.

We investigated the effects of PL on apoptosis and autophagy in MC-3 and HSC-4. First, the cytotoxicity of PL was determined using an MTT assay, and a significant decrease in cell viability was observed in both oral cancer cell lines at PL concentrations of 2 μM or higher. This finding was similar to that of a previous study in which A549 lung cancer cells were treated with 10 μM PL for 24 h, and cell viability was approximately 60% compared to that in the control group [41]. The findings suggested that PL reduced cell viability in a concentration-dependent manner, even in oral cancer cell lines. In a study on ovarian cancer cell lines, namely A2780, OVCAR3, and SKOV3, with PL for 72 h, in a previous study, the IC_50_ values were found to be 6.18 μM, 6.20 μM, and 8.20 μM, respectively. The results showed a substantial difference from the IC_50_ value of 60.23 μM in normal cells [37]. Similarly, in our study, we found that IC_50_ values for PL in MC-3 and HSC-4 cells were 9.36 μM and 8.41 μM, respectively. This indicates the high anticancer effects of PL in oral cancer cell lines as well as in the cancer cell lines mentioned in previous studies. Further, PL is known to hardly affect normal gastric cells compared to the 60–80% inhibition in gastric cancer cell proliferation rate, reported earlier, due to PL [16]. These findings collectively indicate that PL exhibits selective cancer cell-killing, with little effect on normal cells.

Structural changes occur in cells during apoptosis, resulting in distinct morphological characteristics, such as DNA fragmentation, chromatin condensation, and nuclear fragmentation [22,23]. In this study, using DAPI staining, we observed that apoptotic cells increased with an increase in the concentration of PL (0, 4, and 8 μM) in MC-3 and HSC-4 cells. Early apoptotic, late apoptotic, and necrotic cells can be distinguished by annexin V/PI staining [42]. The current study found that the proportion of apoptotic annexin V-positive cells increased in a concentration-dependent manner in the PL-treated group, indicating that PL induced apoptosis in oral cancer cells. Similar to the results of this study, a previous study had reported that the proportion of apoptotic body and apoptosis increased in a concentration-dependent manner when MDA-MB-231, a human breast cancer cell, was treated with PL at concentrations of 0, 5, 10, 15, 20, and 25 μM [43]. In addition, when human gastric cancer cells, namely SGC-1901, BGC-823, and KATO Ⅲ, were treated with PL at concentrations of 0, 5, 10, and 15 μM, the proportion of apoptosis in all three gastric cell lines was identified to have increased significantly in a concentration-dependent manner [16]. The above data supported our current study and suggested that PL indeed reduced the cell viability of MC-3 and HSC-4 through apoptosis.

Apoptosis involves both intrinsic and extrinsic pathways. Mitochondria regulate the intrinsic pathway, whereas death receptors regulate the extrinsic pathway [44]. The ratio of Bax/Bcl-2 protein expression is an essential factor in the mitochondria-mediated apoptotic pathway [45]. The current study found that the Bax/Bcl-2 ratio was increased 2.08-fold and 3.69-fold in MC-3 and HSC-4 cells, respectively, in the 8 μM PL-treated group compared to the control group. Moreover, the level of cleaved PARP was significantly increased by PL in both oral cancer cell lines.

In addition to apoptosis, autophagy plays a crucial role in cancer treatment. The present study identified that PL-induced autophagy is associated with autophagy factors, such as p-mTOR, LC3, and Beclin-1, in oral cancer cell lines. Autophagy plays a dual role in cancer, namely progression and inhibition; the effects vary according to the cell type and stimulation [46]. In this study, cell viability was confirmed following the pretreatment of both MC-3 and HSC-4 cells with 3-MA and HCQ. Here, in both cell lines pretreated with 3-MA, an early-stage inhibitor of autophagy, did not show a significant difference. However, pretreatment with HCQ, a late-stage inhibitor of autophagy, decreased the viability of MC-3 cells, thereby implying a cytoprotective role in the late stages of autophagy. This finding demonstrated that inhibition of autophagy is an effective strategy for improving the anticancer effects of PL in MC-3 oral cancer cell lines.

JNK, a member of the MAPK family, is activated by several cellular stressors and cytokines. It plays a pivotal role in signaling cascades that perform various biological functions and exhibit physiological effects under both normal and pathological conditions [47]. JNK is activated by cellular stress and is closely associated with apoptosis [48]. A previous study found the activation of JNK to be associated with PL-induced apoptotic cell death in HCT116 colon cancer cells [49]. Moreover, the JNK-mediated pathway has been reported to regulate the balance between apoptosis and autophagy in response to genotoxic stress [50]. MAPKs, such as JNK, are involved in the induction of autophagy by phosphorylating Bcl-2 and disrupting the Bcl-2/Beclin-1 complex [51]. The current study found that PL treatment activated the expression of ERK, JNK kinase, and p38. ERK activation has been associated with autophagy induction and there is accumulating evidence that ERK can be activated by oxidative stress, various oncogenes, growth factors and certain anticancer drugs [52]. When JNK was inhibited by SP600125 (a JNK inhibitor), apoptosis and autophagy levels were found to be decreased in MC-3 cells, whereas cell viability increased. Protein levels of cleaved PARP and Bax decreased in the presence of SP600125 when MC-3 cells were treated with PL, whereas the protein expression level of Bcl-2 increased. Thus, SP600125 successfully inhibited the activity of JNK via the protein expression of p-JNK. Furthermore, LC3, an autophagy-associated factor, significantly decreased in the presence of SP600125 when MC-3 cells were treated with PL. This finding implied that pharmacological inhibition of JNK inhibited autophagy and that JNK played a vital role in regulating the balance between apoptosis and autophagy. The role of JNK is similar to that reported in a previous study, in which the pharmacological inhibition of JNK substantially inhibited autophagy in mesenchymal stem cells [53]. Taken together, the results suggested that apoptosis and autophagy are mediated by JNK activation in PL-treated MC-3 cells. This study identified that apoptosis and autophagy were induced simultaneously by PL in human oral cancer cells, and that induced autophagy had a cytoprotective effect in MC-3 cells. This could be attributed to the activation of protective autophagy to adapt to stressful conditions and protect cells from death. Overall, the data suggested that inhibition of cytoprotective autophagy in MC-3 cells substantially strengthened the anticancer effect of PL. Further studies would be required to determine the anticancer effects of PL on oral cancer in vivo. If the efficacy of PL in vivo targeting oral cell lines is verified, it could be used as a powerful natural agent by providing experimental evidence for anticancer activity.

## 5. Conclusions

In summary, our study revealed that PL exerts an inhibitory effect by suppressing the growth of MC-3 and HSC-4 human oral cancer cell lines by inducing apoptosis through modulation of the MAPK signaling pathway. Besides inducing apoptosis, PL extensively leads to autophagy in oral cancer cell lines. Furthermore, the pharmacological blockade of autophagy activation by HCQ, a late-stage inhibitor of autophagy, significantly improved PL-induced apoptosis in MC-3 cells, suggesting cytoprotective effects of PL-induced autophagy. Additionally, pharmacological inhibition of JNK by SP600125, a JNK inhibitor, not only significantly reduced apoptosis in MC-3 cells but also inhibited autophagy. Collectively, the study findings suggested that combining an autophagy inhibitor with PL treatment can exert effective anticancer properties in oral cancer cells by inducing apoptosis as well as cytoprotective autophagy via the MAPK signaling pathway.

## Data Availability

Not applicable.
